# Changes in total body bone mineral density following a common bone health plan with two versions of a unique bone health supplement: a comparative effectiveness research study

**DOI:** 10.1186/1475-2891-10-32

**Published:** 2011-04-14

**Authors:** Joel E Michalek, Harry G Preuss, Harry A Croft, Patti L Keith, Samuel C Keith, Monika Dapilmoto, Nicholas V Perricone, Robert B Leckie, Gilbert R Kaats

**Affiliations:** 1 Department of Epidemiology and Biostatistics, University of Texas Health Science Center at San Antonio, San Antonio, TX., USA; 2 Department of Physiology, Georgetown University Medical Center, Washington, DC USA; 3 Croft Research Group, San Antonio, TX. USA; 4 Integrative Health Technologies, Inc., 4940 Broadway, San Antonio, Texas, 78209 USA; 5 Michigan State University College of Human Medicine, East Lansing, Michigan USA; 6 Business and Healthcare Consultants, San Antonio, TX. USA

## Abstract

**Background:**

The US Surgeon General's Report on Bone Health suggests America's bone-health is in jeopardy and issued a "call to action" to develop bone-health plans that: (1) improve nutrition, (2) increase health literacy and, (3) increase physical activity. This study is a response to this call to action.

**Methods:**

After signing an informed consent, 158 adults agreed to follow an open-label bone-health plan for six months after taking a DXA test of bone density, a 43-chemistry blood test panel and a quality of life inventory (AlgaeCal 1). Two weeks after the last subject completed, a second group of 58 was enrolled and followed the identical plan, but with a different bone-health supplement (AlgaeCal 2).

**Results:**

There were no significant differences between the two groups in baseline bone mineral density (BMD) or in variables related to BMD (age, sex, weight, percent body fat, fat mass, or fat-free mass). In both groups, no significant differences in BMD or related variables were found between volunteers and non-volunteers or between those who completed per protocol and those who were lost to attrition.

Both groups experienced a significant positive mean annualized percent change (MAPC) in BMD compared to expectation [AlgaeCal 1: 1.15%, *p *= 0.001; AlgaeCal 2: 2.79%, *p *= 0.001]. Both groups experienced a positive MAPC compared to baseline, but only AlgaeCal 2 experienced a significant change [AlgaeCal 1: 0.48%, *p *= 0.14; AlgaeCal 2: 2.18%, *p *< 0.001]. The MAPC in AlgaeCal 2 was significantly greater than that in AlgaeCal 1 (*p *= 0.005). The MAPC contrast between compliant and partially compliant subjects was significant for both plans (*p *= 0.001 and *p *= 0.003 respectively). No clinically significant changes in a 43-panel blood chemistry test were found nor were there any changes in self-reported quality of life in either group.

**Conclusions:**

Following The Plan for six months with either version of the bone health supplement was associated with significant increases in BMD as compared to expected and, in AlgaeCal 2, the increase from baseline was significantly greater than the increase from baseline in AlgaeCal 1. Increased compliance was associated with greater increases in BMD in both groups. No adverse effects were reported in either group.

**Trial Registration:**

ClinicalTrials.gov NCT01114685

## Background

In its 2004-2009 Strategic Plan [1] NIH's Office of Dietary Supplements seeks to stimulate research assessing the effects of dietary supplements on biomarkers associated with chronic diseases, optimal health and improved performance. One important biomarker meeting this goal is BMD, often viewed as the "gold standard" for assessing bone health. In the same year (2004), the *Surgeon General's Report on Bone Health *[2] reported that by 2020 half of all American citizens older than 50 will be at risk for fractures, and that there is a bone health crisis in America due to increasingly sedentary lifestyles, absence of current information about bone health, and inadequate nutrition. The Surgeon General (SG) recommended that people of all ages ensure they are getting the recommended amounts of calcium and vitamin D and that supplementation may be helpful. Pointing out that people are never too young or too old to improve their bone health, the SG issued a "call to action" for the development of bone health programs incorporating three components: (1) improved nutrition, (2) improved health literacy, and (3) increased physical activity,.

More recently, the current emphasis on CER studies suggests they may provide an important methodology for responding to the SG's call to action. As defined by The American College of Physicians, CER is the evaluation of the relative clinical effectiveness, safety, and cost of two or more medical services, drugs, devices, therapies, or procedures used to treat the same condition [3]. CER studies not only compare different drugs, but healthcare plans that include lifestyle modifications such as diet and physical activity, and complementary and alternative therapies that are often initiated without physician input [4]. CER is a marked departure from the past research models that have focused on demonstrating superiority over placebos, instead of comparing the relative efficacy and safety of new therapeutic interventions [5]. This has often has led to approval of a number of "me too" interventions that ultimately rely on a company's marketing skills as opposed to demonstration of superior safety and efficacy. Publication of the results of CER studies will also require a paradigm shift in the scientific community where, traditionally, the use of placebo or control groups in studies significantly increases the chances for publication [6].

The purpose of this CER study was to compare changes in BMD in two bone health plans with each other and with age- and gender-adjusted expected changes.

## Methods

The study was approved by RCRC Institutional Review Board http://www.RCRCIRB.com, Austin, TX, Protocol number 1252006. It was funded by a small nutritional company with a limited budget that initially sought to examine the safety and efficacy of a bone-health plan using an open-label protocol under "real world" conditions approximating those in which consumers were likely to follow either Plan. Additionally, advising subjects that they had a 50-50 chance of receiving an inactive placebo for six months was thought to increase the difficulty in recruiting subjects, particularly highly motivated subjects who were seeking to improve their bone health, thus creating volunteer biases. The decision was also influenced by a desire to use available funds to increase the number of subjects in the treatment group in order to examine volunteer bias and conduct sub-group compliance analyses.

During the study, new information became available suggesting that The Plan might be enhanced by making changes in the nutritional composition of the bone-health supplement. Upon receipt of the ending data, a second study was commissioned to retain the physical activity and health literacy component, but to alter the bone-health supplement with different kinds and amounts of some of the bone-health ingredients.

### The Bone-Health Plans

To provide the improved nutrition component of the SG's recommendations, the two groups were provided with the bone-health supplements shown in Table 1. Both formulas were analyzed by Exova Labs, Chicago, IL for confirmation of nutrient levels and lack of heavy minerals and other contaminating ingredients. Calcium, magnesium and other minerals were validated by Advanced Labs, Salt Lake City, UT. AlgaeCal (AC) is a plant-sourced form of calcium made by milling whole, live-harvested sea algae found on the South American coastline. In addition to calcium, this algae contains 13 other minerals known to play a role in bone health, including magnesium, boron, silica, manganese, copper, vanadium and strontium. A recent in vitro study demonstrated superiority over the two most commonly used calcium salts, calcium carbonate and calcium citrate. Cultured human osteoblast cells (hFOB 1.19) were treated with either AC, calcium carbonate or calcium citrate. Alkaline phosphatase activity was significantly increased with AC treatment when compared to control, calcium carbonate or calcium citrate (4.0, 2.0 and 2.5-fold, respectively). Proliferating cell nuclear antigen expression (immunocytochemical analysis), DNA synthesis (4.0, 3.0 and 4.0 fold, respectively) and Ca2+ deposition (2.0, 1.0 and 4.0 fold, respectively) were significantly increased in AC treated cells when compared with control, calcium carbonate, or calcium citrate treatment. AC treatment significantly reduced the H2O2-induced oxidative stress when compared to calcium carbonate or calcium citrate (1.5, 1.4 fold, respectively). This earlier study demonstrated that AC exhibited unique properties compared to calcium carbonate or calcium citrate on a cellular level which suggests the need for human intervention studies such as the present study [7]. Furthermore, safety and toxicological investigations were conducted using AC and demonstrated its broad spectrum safety [8].

**Table 1:** Components of two versions of the bone-health plan provided to subjects in Grp 1 and Grp 2

Ingredient or Component	Grp 1	Grp 2
Pedometer-based activity program	Yes	Yes
Health Literacy Information	Yes	Yes
Strontium Citrate (mg)	680	680
AlgaeCal Bone-health Supplement	2,400	2,520
Trace Minerals in AlgaeCal (mg)	1,608	1,688
Calcium (mg)	720	756
Magnesium (mg)*	72	75
Magnesium from magnesium carbonate (mg)	0	275
Vitamin D-3 (IUs of Cholecalciferol)	800	1,600
Vitamin K-2 as MK-4 (mg)	1.5	0
Vitamin K-7 as MK-7 (mcg)	0	100
Boron (mg)	0	3
Vitamin C (mg)	0	50

*72 mg naturally occurring plus magnesium carbonate

To provide a health literacy component, with permission from the author, subjects were provided with reprints from Chapters 5, 8, 9 & 10 of a previously published book [9]. These chapters provided information on bone density, and on the pedometer-based physical activity program. Calorie estimation charts and glycemic load tables of over 300 common foods designed to increase the quality of carbohydrate intakes were also included [9].

To increase physical activity levels, subjects were asked to wear a pedometer during their waking hours and to record and track their daily activity levels using the charts and graphs provided in their health literacy booklet. They were also asked to follow the instructions for personalizing the pedometer program to their personal goal weight and stride length. In addition to the potential benefit for bone health, two reviews have suggested that the use of pedometers can lead to increased physical activity levels and significant health benefits [10,11]. The Digi-Walker pedometer (HealthTech Products, LLC, San Antonio, TX) used in this study is generally considered among the most reliable and valid of pedometers available [12].

### Subjects

**Figure 1:**
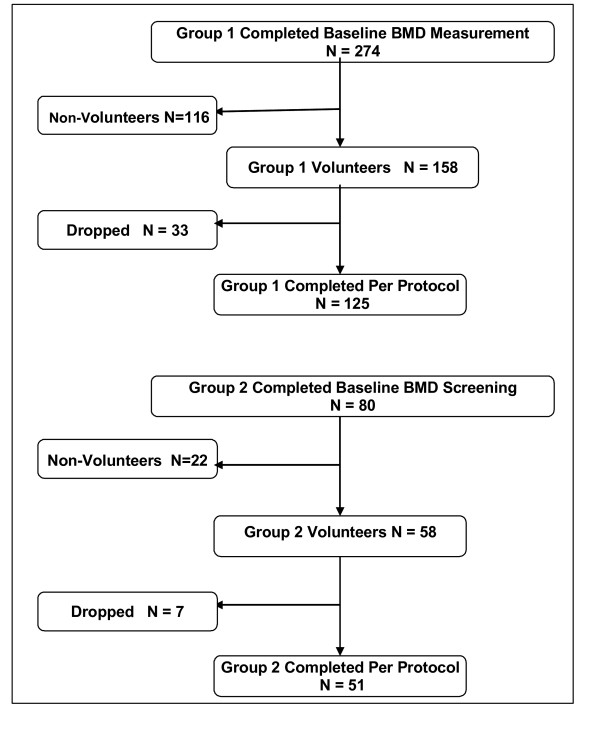
**Flow of participants through the trial for both study groups**.

As shown in Figure 1, a total of 274 adults aged 18-85 were contacted from the investigators' DXA database and from participants in a local health fair and were invited to complete a bone density test and to have the study explained to them prior to enrolling (AC-1). Of these 274 potential subjects, 158 agreed to participate and certified that they had reviewed the Informed Consent with their personal healthcare provider or physician and that they had no medical conditions that would preclude their participation. However, pregnant and lactating women were excluded irrespective of this certification. Subjects were asked to refrain from taking other bone-health supplements during the study. Of these 158 adults, 125 ultimately completed the study PP, which included providing weekly tracking data to Integrative Health Technologies' research center in San Antonio, TX

Upon completion of the study in which subjects used AlgaeCal 1, (AC-1) a second group, AlgaeCal 2 (AC-2) of 80 adults followed the identical pre-enrollment procedure as AC-1. Again, subjects and research technicians were blinded with regard to the subjects' baseline test results. Of this total, 58 agreed to participate, 51 of whom ultimately completed the study PP. Although subjects in this second group followed the same Plan, they took the revised version of the bone health supplement shown in Table 1.

Considerable effort was devoted to maintaining identical conditions for both groups in order to permit a comparison of the effects on mean BMD of the two versions of the bone health supplement. To encourage candid reporting for acquiring dose-related and compliance comparisons, subjects in both groups were paid a "reporting fee" of $2.00/day for providing daily reports of supplement usage and side effects. Throughout the study, subjects were repeatedly reminded that this fee was not an "incentive" for taking the product, but rather was for the purpose of obtaining candid information on the effects of different levels of adherence to the bone-health plan. Payment of the fee was contingent upon reporting tracking information weekly and completing the ending tests. Thus, compliance was used as an additional measure of efficacy on the assumption that if The Plan was efficacious, compliant subjects would outperform partially compliant subjects.

### Outcome Measures

To assess efficacy, changes in BMD were measured at baseline and six months from baseline using dual-energy X-ray absorptiometry (DXA) total body scans (GE Lunar Prodigy, LUNAR Corporation, Madison, WI, USA). Longitudinal precision was monitored for all measurements using the same bone density phantom provided by the manufacturer. To compare changes in mean BMD over different study periods, data were converted to MAPC. For calculation of expected changes, we used age- and gender-adjusted norms from: GE Lunar, our database of over 26,000 total body measurements, and data from the National Osteoporosis Foundation [13]. We used an expected change for women under 40 yrs of +0.1%, for 41-55, -0.5%/yr, 56 and older, -1.0%/yr and used half these amounts for males. This may be a somewhat conservative estimate in view of population-based longitudinal studies suggesting that, starting at age 40, there is minor, but significant, annual bone loss [14] that increases to 0.5% to 0.9% a year in perimenopausal women [15-18], to above 1% after menopause [17,18] after which the decline remains about 1% [14,19,20]. Other studies suggest after midlife there is an age-related yearly loss of bone in both sexes of 1% [21] which is accelerated to 2% for up to 14 years in women around the age of menopause [22]. In men, a small loss is detected in 40-year olds [14] that increases to a ~0.8% per year into old age [14,19-21]. More recently, another review has suggested that women will lose 35% to 39%, men 17%-19%, of lifetime bone loss after achieving peak bone mass at ages 30-40 years [23], changes that are consistent with the previously cited studies. Additionally, and perhaps an even more conservative estimate, comparisons were also made using ±0%/yr for all subjects in both groups and sub-groups.

To evaluate safety, daily tracking self-reports, the 43-item blood chemistry panel, and the 50-item Quality of Life inventory [24] shown in Tables 2 and 3 were administered to all study participants at baseline and at the end of six months.

**Table 2:** Comparison of Baseline Demographics

AlgaeCal 1 n = 125		AlgaeCal 2 n = 51	
**Demographic**	**Mean ± SD**	**Mean ± SD**	**P-Value**

**Females**	86.4%	78.4%	0.341
**Age (years)**	55.2 ± 11.2	56.7 ± 13.2	0.463
**Weight (lbs)**	153.6 ± 38.8	167.7 ± 55.0	0.709
**% Body Fat**	39.9% ±10.0%	35.9% ±10.2%	0.591
**Fat Mass**	63.0 ± 26.6	63.5 ± 38.3	0.938
**Fat Free Mass**	97.8 ± 21.1	104.2 ±23.9	0.082

**Table 3:** Changes in blood chemistries in 126 subjects with study groups AlgaeCal 1and 2 Combined

Chemistry	Normal Range	Baseline Mean	Ending Mean	Change	*P*-Values
**TRIGLYCERIDES**	**<150 mg/dL**	111.4	114.7	3.26	0.395
**CHOLESTEROL, TOTAL**	**125-200 mg/dL**	193.3	196.7	3.38	0.171
**HDL CHOLESTEROL**	**> or = 40 mg/dL**	58.2	58.8	0.59	0.388
**LDL CHOLESTEROL**	**<130 mg/dL**	112.8	114.9	2.13	0.354
**CHOL/HDLC RATIO**	**< or = 5.0**	3.5	3.6	0.02	0.705
**GLUCOSE**	**65-95 mg/dL**	96.9	95.8	-1.12	0.558
**UREA NITROGEN (BUN)**	**7-25 mg/dl**	14.4	14.8	0.44	0.163
**CREATININE**	**0.50-1.20 mg/dL**	0.86	0.88	0.02	0.002
**BUN/CREATININE RATIO**	**6-22**	17.1	17.1	-0.05	0.896
**SODIUM**	**135-146 mmol/L**	140.0	140.2	0.13	0.476
**POTASSIUM**	**3.5-5.3 mmol/L**	4.3	4.4	0.02	0.502
**CHLORIDE**	**98-110 mmol/L**	105.0	104.7	-0.29	0.001
**CARBON DIOXIDE**	**21-33 mmol/L**	24.4	25.1	0.79	0.132
**CALCIUM**	**8.6-10.2 mg/dL**	9.38	9.53	0.15	0.000
**PROTEIN, TOTAL**	**6.2-8.3 g/dL**	7.0	7.0	-0.02	0.586
**ALBUMIN**	**3.6-5.1 g/dL**	4.3	4.3	0.02	0.311
**GLOBULIN**	**2.2-3.9 g/dL**	2.7	2.7	-0.04	0.080
**ALBUMIN/GLOBULIN RATIO**	**1.0-2.1 mg/dL**	1.6	1.7	0.03	0.032
**BILIRUBIN, TOTAL**	**0.2-1.2 mg/dL**	0.6	0.6	-0.02	0.392
**ALKALINE PHOSPHATASE**	**33-115 U/L**	68.7	69.3	0.64	0.629
**AST**	**10-35 U/L**	20.7	18.5	-2.21	0.005
**ALT**	**6-40 U/L**	20.4	18.8	-1.61	0.163
**WHITE BLOOD CELL COUNT**	**3.8-10.8 Thousand/uL**	5.9	5.9	0.08	0.475
**RED BLOOD CELL COUNT**	**3.80-5.10 Million/uL**	4.5	4.5	-0.01	0.706
**HEMOBLOBIN**	**11.7-15.5 g/dL**	13.6	13.6	0.01	0.719
**HEMATOCRIT**	**35.0-45.0%**	40.4	40.5	0.11	0.467
**MCV**	**80.0-100.0 fL**	90.5	90.9	0.39	0.024
**MCH**	**27.0-33.0 pg**	33.7	33.6	-0.07	0.254
**MCHC**	**32.0-36.0 g/dL**	30.5	30.6	0.06	0.266
**RDW**	**11.0-15.0%**	13.8	13.7	-0.11	0.106
**PLATELET COUNT**	**140-400 Thousand/uL**	249.4	247.4	-2.05	0.339
**ABSOLUTE NEUTROPHILS**	**1500-7800 cells/uL**	3,460	3,534	74.0	0.442
**ABSOLUTE LYMPHOCYTES**	**850-3900 cells/uL**	1,858	1,821	-37.0	0.231
**ABSOLUTE MONOCYTES**	**200-950 cells/uL**	373.6	390.3	16.8	0.050
**ABSOLUTE EOSINOPHILS**	**15-500 cells/uL**	146.8	168.8	22.0	0.014
**ABSOLUTE BASOPHILS**	**0-200 cells/uL**	25.5	25.9	0.35	0.795
**NEUTROPHILS**	**55-70%**	58.0%	58.5%	0.01	0.482
**LYMPHOCYTES**	**20-40%**	32.3%	31.5%	-0.01	0.119
**MONOCYTES**	**2-8%**	6.7%	6.8%	0.00	0.498
**EOSINOPHILS**	**1-4%**	2.6%	2.9%	0.00	0.033
**BASOPHILS**	**0.5-1.0%**	0.5%	0.4%	0.00	0.678
**TSH W/REFLEX TO FT4**	**0.40-4.50 mIU/L**	5.9	9.0	3.10	0.175
CARDIO CRP*		3.9	4.8	0.90	0.868

* <1.0 = Low Cardiovascular Risk, 1.0-3.0 = Average Risk, 3.1-10.0 High Risk, > 10.0 = Repeat test

### Compliance

To obtain a compliance rating, the research technician who had the most frequent contact with subjects rated the subjects' compliance with the protocol using a 5-point scale with "5" indicating "highly compliant" and "1" indicating "not at all compliant" with the protocol. This judgment was based on the subject's report of the amount of product he/her consumed, the number of steps reported on pedometer usage, whether or not they had acquired any information from the health literacy component, and her evaluation of the reliability of the subject's self-reported data based on her contact with the subject over the 6-month study period. Upon completion of the technician's ratings, one of the investigators (GRK) reviewed the data from the subjects' daily tracking forms and post-study anonymous questionnaires and compared it to the previous ratings. Finally, joint subjective evaluation of the subjects' overall compliance was made. Less than 10% of the technicians' ratings were changed as a function of this joint review. When making these ratings, both the technician and the investigator were blinded with respect to the subject's BMD measurements.

### Statistical Methods

To address concerns of bias in industry-sponsored research, we provided an independent academic statistician who conducted all statistical analyses. He served as the Principal Investigator, had full access to all study data and source documents, and took responsibility for the integrity of the data and the accuracy of the data analysis.

Continuously distributed data were summarized with the mean and standard deviation, and binary outcomes were summarized with counts and percents. AC-1 and AC-2 were contrasted on MAPC with analyses of covariance with adjustment for age and sex. Group contrasts with regard to binary outcomes were made with Pearson's chi-square. All statistical testing was 2-sided with a significance level of 5%. SAS Version 9.1.3 for Windows (SAS Institute, Cary, North Carolina) was used throughout.

## Results

As shown in Table 2, there were no significant differences between the groups in baseline BMD or in baseline variables related to BMD, nor were there any differences within these variables between subjects who chose not to enroll and those who completed PP, or between those who enrolled, but dropped, as compared to those who completed PP.

**Figure 2:**
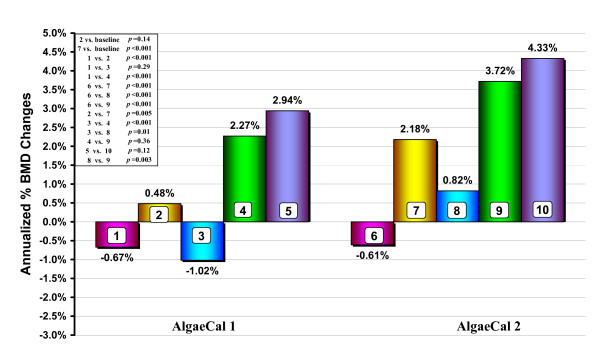
**Comparisons of mean annualized changes in bone mineral density from baseline in two groups, AlgaeCal 1 and AlgaeCal 2, following the same bone-health plan, but with different versions of an enhanced plant-sourced form of calcium**. AlgaeCal 1: 1 = Expected Changes (adjusted for age & gender), 2 = Change from Baseline, 3 = Changes in Partially Compliant Subjects, 4 = Changes in Compliant Subjects, 5 = Changes in Compliant Subjects Over-expected (1 + 4). AlgaeCal 2: 6 = Expected Changes (adjusted for age & gender), 7 = Change from Baseline, 8 = Changes in Partially Compliant Subjects, 9 = Changes in Compliant Subjects, 10 = Changes in Compliant Subjects Over-expected (6 + 10).

Figure 2 shows bar-graphed representations of expected and actual MAPC in BMD for both study groups, compliant and partially-compliant sub-groups, and over-expected changes in the two study groups. The MAPC in BMD:

▪ minus the expected MAPC was greater than zero in AC-1 [1.15% (3.62), *p *< 0.001] and in AC-2 [2.79% (3.57), *p *< 0.001];

▪ increased from baseline in AC-2, [2.18% (3.58), p < 0.001], but not in AC-1, [0.48% (3.64), *p *= 0.14];

▪ from baseline was greater in AC-2 [2.18% (3.58), than in AC-1 [0.48% (3.64), *p *= 0.005);

▪ was greater in compliant subjects than in partially compliant subjects in AC-1 [2.27% (3.62) vs. -1.02% (2.92), *p *< 0.001], and in AC-2 [3.72% (3.96) vs. 0.82% (2.58), *p *= 0.003];

▪ of 3.72% in compliant subjects in AC-2 was greater than in the 2.27% increase in AC-1, but this difference failed to reach statistical significance (*p *= 0.12);

▪ among partially compliant subjects, the 0.82% increase in AC-2 was significantly greater than the -1.02% decrease in AC-1 (*p *= 0.005);

▪ among compliant subjects the over-expected increase in AC-2 [4.30% (3.98)] was greater than AC-1 [2.27% (3.62)], but this difference failed to reach statistical significance (*p *= 0.12).

With regard to the measures of safety, there were no significant differences in blood chemistries between the two study groups at baseline or end-of-study. Therefore, their data were combined and are shown in Table 3. Although the baseline/ending changes in 6 of the 43 blood chemistries were statistically significant, in no instance did the average change exceed normal ranges. Thus, changes within normal ranges were considered clinically insignificant. With regard to the QOL, there were no significant baseline/ending changes in any of the 50 items or in the total scale scores, as shown in Table 4.

**Table 4:** Changes in Blood Chemistries for 126 Subjects completing the test battery at baseline and end-of-study

RATING SCALE	Baseline Mean	Change from Baseline	Repeated Measures *P *Levels	RATING SCALE	Baseline Mean	Change from Baseline	Repeated Measures *P *Levels
0 = Not a problem 1 = A MINOR problem				0 = Not a problem 1 = A MINOR problem			
2 = A MAJOR problem 3 = A SEVERE problem				2 = A MAJOR problem 3 = A SEVERE problem			
Average Total Score	0.289	-0.001	*0.958*	25 Lupus*	0.031	0.000	*N/A*
01 Headaches	0.328	0.023	*0.670*	26 Irregular heartbeat	0.185	-0.040	*0.277*
02 Irritable bowel syndrome	0.215	0.059	*0.171*	27 Shortness of breath	0.131	-0.001	*1.000*
03 Arthritis	0.512	-0.015	*0.725*	28 Constipation or diarrhea	0.397	0.046	*0.448*
04 Premenstrual syndrome	0.156	0.025	*0.435*	29 Stomach gas or indigestion	0.411	-0.003	*0.774*
05 Recurring sinus infections	0.260	-0.038	*0.355*	30 Feeling weak	0.290	0.002	*1.000*
06 Tension fatigue syndrome	0.305	0.067	*0.319*	31 Eating too rapidly	0.346	-0.056	*0.150*
07 Recurrent anxiety	0.285	-0.010	*0.854*	32 Eating after being full	0.359	-0.051	*0.251*
08 Recurrent depression	0.275	0.000	*1.000*	33 Embarrassed about overeating	0.122	0.046	*0.202*
09 Insomnia	0.481	0.015	*0.815*	34 Depressed over eating habits	0.160	0.023	*0.614*
10 Low self esteem	0.260	-0.046	*0.305*	35 Depressed about my weight	0.374	0.008	*0.887*
11 Binge eating	0.176	0.092	*0.064*	36 Difficult to stop eating	0.214	0.002	*1.000*
12 Chronic tension	0.321	0.008	*0.889*	37 Worrying about the future	0.450	-0.050	*0.624*
13 Lack of energy	0.669	0.003	*1.000*	38 Unable to concentrate	0.481	-0.112	*0.058*
14 Food allergies	0.260	-0.044	*0.258*	39 Forgetfulness	0.588	-0.023	*0.707*
15 Feeling under stress	0.809	-0.008	*0.916*	40 Bad temper or quick to anger	0.244	-0.015	*0.725*
16 Cancer	0.031	0.008	*0.656*	41 Indigestion	0.214	-0.015	*0.696*
17 Prostate problems	0.057	-0.015	*0.158*	42 Diabetes	0.130	-0.031	*0.250*
18 Overeating	0.385	0.035	*0.663*	43 Vomiting	0.000	0.000	*N/A*
19 Stomach pain	0.099	0.084	*0.048*	44 Heartburn	0.208	0.008	*0.858*
20 Back pain	0.649	0.015	*0.797*	45 Esophageal reflux	0.214	-0.037	*0.299*
21 Pain in arms, legs or joints	0.656	-0.008	*0.887*	46 Control over my appetite	0.305	0.000	*1.000*
22 Menstrual pain or problems	0.160	-0.003	*1.000*	47 Ability to relax	0.443	0.015	*0.794*
23 Chest pain	0.085	-0.016	*0.529*	48 Heart disease	0.115	-0.023	*0.319*
24 Dizziness	0.154	0.022	*0.566*	49 Fibromyalgia	0.096	-0.013	*0.259*

*At baseline, only one subject recorded a rating for Lupus and no one, including this subject, recorded any ending rating of Lupus precluding the calculation of a *P *value and making the rating irrelevant. It is our view that most likely the baseline rating was an error.

## Discussion

This CER study was initially designed to compare changes in BMD in a bone health plan that incorporated the three components recommended in the SG's "call to action" (improved nutrition, increased health literacy, and increased physical activity) with expected changes in BMD as reported in non-intervention studies. In addition to the primary outcome measure of changes in BMD, measures of safety, volunteer biases, dropout effects, and effects of compliance were also examined. Upon completion of this initial study, a second study was commissioned to compare the effects of following the same bone-health plan, but with a different version of the calcium bone-health supplement.

Although the sequential design, as opposed to an RCT, posed difficulties in interpreting the data, the results suggests that following the AC-2 plan led to significantly greater increases in BMD than expected and than the AC-1 plan. This conclusion is based on between-group comparisons of the MAPC in: (A) AC-1 as compared to (B) AC-2, an untreated age- and gender-adjusted expected change control group, and (C) between compliant sub-groups in AC-1 and AC-2. Support for The Plan's efficacy is also provided by the within-group comparisons of changes from baseline and between compliant and partially-compliant sub-groups, a finding consistent with an exhaustive meta analysis of 23 trials (n = 41,419) of the effects of supplementation on BMD [25]. These researchers concluded that poor compliance is the major obstacle to obtaining full benefit of supplementation and that compliant subjects doubled their risk reduction, suggesting high compliance is needed to demonstrate the therapeutic efficacy of supplementation. Support for the safety of the AC-1 Plan is provided by the absence of adverse events or changes from baseline in the QOL, daily tracking reports and the 43-chemistry blood panel.

One apparent difficulty in interpreting the findings is the loss of 1.02% of MAPC in the partially-compliant sub-group following the AC-1 plan (bar-graph #3, Figure 2). Although the bar-graph suggests that this sub-group lost more BMD than expected (bar-graph #1), the difference was not statistically significant (*p *= 0.29) suggesting that changes in the AC-1 plan were no different than expected. The most parsimonious explanation for the absence of any change in BMD for the partially compliant sub-group taking AC-1 is that this bone-health plan had no effect on BMD when subjects only partially adhered to The Plan, but did facilitate change among more compliant subjects. Conversely, when following The Plan with AC-2, even the partially-compliant subjects increased their BMD and subjects classified as compliant had greater increases than partially-compliant subjects taking AC-2. Thus, the data suggest that there may be a threshold below which no changes in BMD occur and above which changes do occur. The threshold appears to be between partially compliant and compliant subjects taking AC-1.

### Study Weaknesses and Mitigating Factors

Although these data support a comparative effectiveness interpretation of the superiority of the AC-2 plan, absent a placebo-controlled arm, one could conclude that the increased MAPC from baseline and over-expected was attributable to using a sequential design resulting in unequal subject groups, a placebo effect or invalid expected change data. With regard to the equivalence of the groups, while it was impossible to rule out all potentially confounding variables, increased confidence in the similarity of the two groups was obtained from comparisons of a number of baseline measures associated with changes in BMD. There were no statistical differences between the groups on age, gender and BMD. Nor were there any significant baseline and pre- post-study differences on body composition variables that have been reported to affect BMD [26] (weight, lean mass, % fat, and BMI). Additionally, no differences were found between the groups on the QOL, lipid panel, C-reactive protein, serum calcium or thyroid levels. Of the 43 blood chemistries measured in both groups, only two (platelets and alkaline phosphatase) differed between the groups (*p *< 0.01). In neither group were there differences between volunteers and non-volunteers, nor between those subjects who completed versus those who dropped out. Taken together, these similarities provide considerable evidence that the two groups were reasonably equivalent at baseline.

With regard to placebo effects, it seems implausible to suggest that the reported changes in BMD were the result of placebo effects, particularly in view of a number of studies comparing changes in BMD that showed virtually no change in the placebo arms. For example, three randomized double-blinded placebo-controlled studies measuring the effects of strontium ranelate [27-29] found a progressive and linear decrease in BMD in each of three years with a 1% decline after 12 months--a decline virtually identical to the two age-gender adjusted expected changes used in this study.

In contrast to the absence of studies on placebo effects on BMD, a number of studies have suggested that consumption of the multiple nutrients in AC-1 and AC-2 could facilitate increases in BMD. Although we could find no studies on the effects on BMD of strontium citrate used in both formulas (as opposed to the plethora of studies on strontium ranelate), considerable evidence is available on the bone-health effects of the other nutrients in the AC formula--supplemental magnesium, vitamin K-2, [30,31] and calcium and Vitamin D_3 _[32]. Vitamin C has also been reported as an essential nutrient for collagen formation and normal bone development, particularly in older men and women [33,34]. Further support for the increased BMD may be because AC is a plant-sourced supplement and some studies have suggested plant-sourced minerals may be more easily absorbed than non-plant-sourced calcium and minerals [35-39] suggesting that the body was able to use less than 10 percent of the synthetic minerals contained in the most popular brands of multivitamins as opposed to over 80 percent of minerals derived from plant sources. Other studies have also reported positive associations between fruit and vegetable consumption and BMD in elderly adults [40,41], adolescents [42] and children [43].

With regard to the validity of the expected change data, the studies cited above suggest that the annual expected decrease in BMD is closer to -1.0% as opposed to the -0.67% and -0.63% used in this study. Additionally, although some studies have reported that supplementation with vitamin D_3 _and calcium had no effect on the decline of age-related BMD [44,45], the general consensus is that supplementation does result in a lower rate of annual bone loss [26]. These data would suggest that even with supplementation, the expected annualized change in BMD is between the -1.0% and -0.2%, particularly since there is no compelling evidence that supplementation leads to an increase in BMD.

No attempt was made to partition the effects of the three components of The Plan, since the goal of the study was to examine the effectiveness of the plans, not the individual components in the plans. However, the increased MAPC found in AC-2, as compared to AC-1, suggests that the modifications made to the nutritional profile of AC-2, while holding all other components constant, provided additional benefits over and above the benefits provided by the other components of The Plan.

### Study strengths

One strength of this study is that it was conducted in "real world" conditions which maximized the inclusion criteria and minimized the exclusion criteria by enrolling adults of all age, gender and ethnicity, which increases confidence that the results could be generalized to populations that are most likely to use the product. Other strengths include the well established reliability and validity of DXA measurements of BMD, the analysis and absence of evidence of volunteer and attrition biases, baseline similarities between the two treatment groups, consistency of the expected within-groups differences in compliance, and the experience levels of the testing and research technicians.

With regard to safety, the use of pre- and post-study QOL inventories and independently-measured blood chemistries completed by both groups at baseline and end-of-study contribute to the safety of the study, as does the absence of reported adverse effects on these measures and on the daily tracking forms.

## Conclusions

Compared to an initial formulation, using the revised AC nutritional supplement, AC-2, with additional levels and types of nutrients, while holding all other components of The Plan constant, was associated with significantly greater increases in mean bone density. These increases were significantly greater than baseline BMD and as compared to age- and gender-adjusted expected changes. No evidence was found of adverse side effects, volunteer bias, drop-out bias, or differences between the age and gender of the participant. Additional support for the efficacy of AC-2 was found by significant differences between compliant and partially compliant participants, suggesting a does-related effect. Notwithstanding the absence of an RCT, these findings warrant further study in view of the unusual increases in BMD in both study groups. It is a marked departure from previous studies in which the decline in BMD has been found to be slowed or, at best, maintained.

## Abbreviations

BMD: Total Body Bone Mineral Density; CER: Comparative Effective Research; DXA: Total Body Dual-energy X-ray Absorptiometry; MAPC: Mean Annualized Percent Change in BMD; Plan (or The Plan): the plan under study, incorporating components to promote health literacy, increased physical activity, and improved nutrition; PP: per protocol; QOL: a 50-item Quality of Life questionnaire; SG: United States Surgeon General.

## Competing interests

Dr. Kaats holds an equity position in Integrative Health Technologies, Inc., a public company that provided some of the funding for this study. All other authors declare that they had no competing interests.

## Authors' contributions

JEM was the principal investigator, secured and audited all study data, conducted all of the statistical analyses, and contributed significantly to the preparation and submission of the manuscript. HGP, HAC, NJP contributed to the study design, data interpretation and manuscript review and preparation. PLK edited, revised, and proofed the final manuscript. SCK and MD recruited and enrolled subjects, had weekly contact with all subjects, conducted all DXA testing, provided subjects with requisitions to conduct off-site blood testing, and reviewed and explained the informed consent form. RBL aided in the interpretation of the data and in the preparation, editing and revising of the manuscript. GRK served as the on-site supervisor of all research activities and contributed significantly to the design of the study and the preparation, editing, and revisions of the manuscript. All authors read and approved the final manuscript.
